# Is the gut microbiome of insects a potential source to meet UN sustainable development goals to eliminate plastic pollution?

**DOI:** 10.1111/1758-2229.13166

**Published:** 2023-09-08

**Authors:** Ruqaiyyah Siddiqui, Naveed Ahmed Khan

**Affiliations:** ^1^ College of Arts and Sciences American University of Sharjah Sharjah United Arab Emirates; ^2^ Department of Medical Biology, Faculty of Medicine Istinye University Istanbul Turkey; ^3^ Department of Clinical Sciences, College of Medicine University of Sharjah Sharjah United Arab Emirates

## Abstract

As insects such as cockroaches can endure high radiation, flourish in unsanitary circumstances, thrive on germ‐infested feed, and can even digest the organic polymer cellulose, the gut microbiota of these species likely produces enzymes contributing to their ability to digest a variety of materials. The use of cockroaches as a bio‐resource to eliminate plastic is discussed. We explore whether species such as cockroaches are a potential bio‐resource to eliminate plastic pollution and contribute to the sustainable development goals adopted by the United Nations as well as the global community to reduce and/or eliminate plastic pollution.

## LEARNING FROM OTHER SPECIES

**FIGURE 1:**
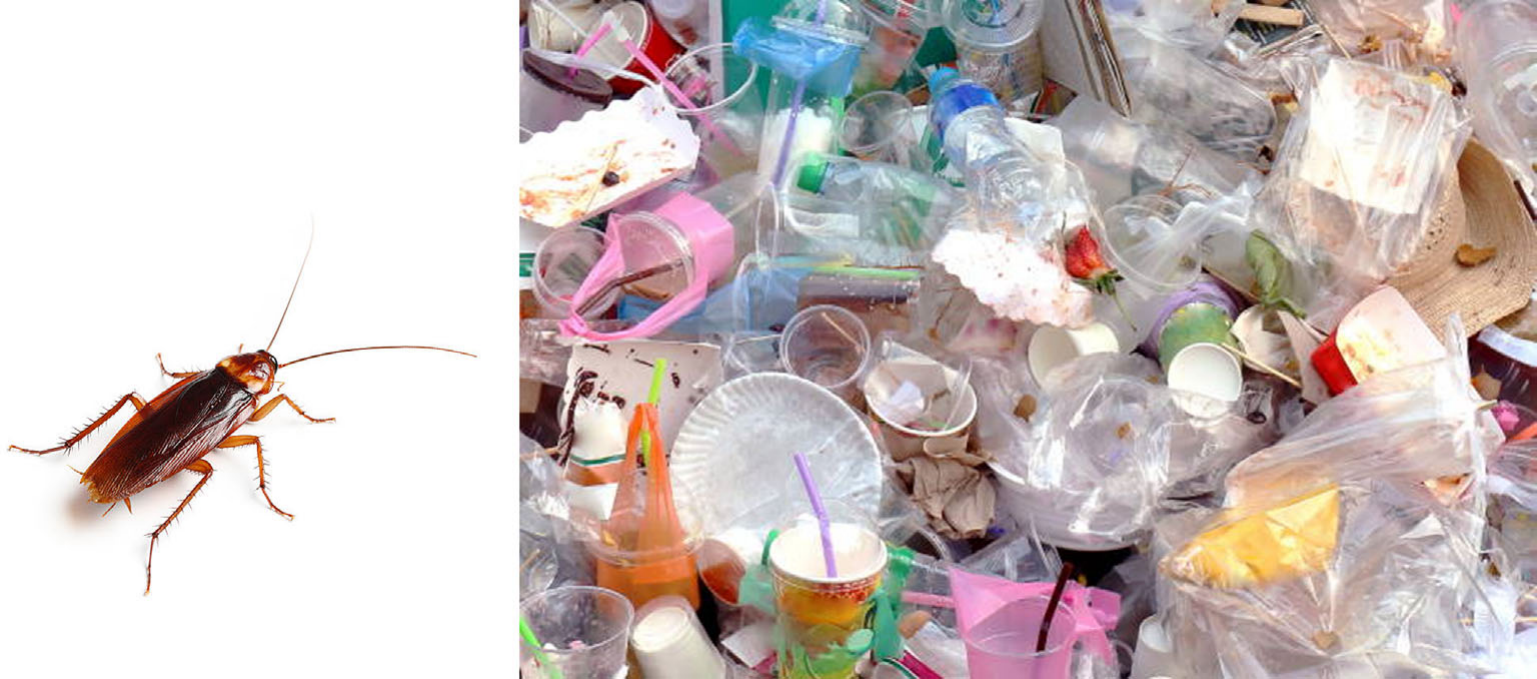
Species such as cockroaches could offer the solution to meet UN sustainable development goals to eliminate plastic?

Insects such as cockroaches can withstand high levels of radiation (up to 10,000 rads) and are one of the hardiest insects on the planet (Akbar et al., 2019; Wharton & Wharton, 1959). They reside in unsanitary environments, can survive for up to a month without food and/or utilise a predominantly germ‐infested diet, and are among the few species to survive since the Carboniferous period between 354 and 295 million years ago (Akbar et al., 2019; Wharton & Wharton, 1959). Moreover, these insects have been shown to possess remarkable antimicrobial properties (Ali et al., 2017). Cockroaches are omnivorous and versatile in their diets, feeding on an assortment of foods (including bread, leather, starch, paper, skin flakes, hair, dead insects, soiled clothing and lignocellulosic materials), and we observed in our laboratory that they can also feed on various types of plastic (Figure 1; Akbar et al., 2019; Ali et al., 2017) and thus may have the potential to degrade plastic waste. Furthermore, it is recognised that the larval stages of some insects in the orders Coleoptera and Lepidoptera may consume plastic (LeMoine et al., 2020). An example is the larvae of the greater wax moth (*Galleria mellonella*), which were shown to biodegrade low‐density polyethylene (LeMoine et al., 2020). However, a recent report suggested that although *G. mellonella* larvae can consume polylactic acid plastic, some metabolic stress is experienced as a result (Shah et al., 2023). Future research into the molecular mechanisms underlying this biodegradation process may contribute to the development of methods for stress management that would hasten the digestion of plastic by such insects. In another study, the biochemical impact of plastics on various insect models: black soldier fly, mealworm and wax moth larvae were analysed and the data suggested a positive insect‐specific interaction towards certain plastic types, although further work is needed to understand the metabolic impact (Beale et al., 2022). Interestingly, it was revealed that the gut bacteria of earthworms were able to digest and significantly reduce low‐density polyethylene (Lwanga et al., 2018). Of note, cockroaches have been shown to feed on cellulose, a very stable polymer, which is shown to be digested by cockroach enzymes (Weihmann et al., 2015), but the source of such enzymes is not clear. One explanation for this could be due to the unique gut microbiota that they possess (Akbar et al., 2019; Bell et al., 1990; Bertino‐Grimaldi et al., 2013). This is further corroborated by the evidence that some species of cockroaches are known to possess gut microbiota, which can digest cellulose, on the other hand, some species secrete cellulase in their saliva (LeMoine et al., 2020; Wharton & Wharton, 1959). Moreover, it has been suggested that the deployment of such gut bacteria which possess potent lignocellulolytic potential, may be harnessed as sustainable bio‐resource technology for augmented biogas production and utilising waste‐plant biomasses (Show et al., 2022). However, whether plastics are digested by these species and the underlying molecular mechanisms or are excreted as microplastics in their faeces is unclear and needs to be investigated in the future studies. Furthermore, the metabolic health of cockroaches following the ingestion of plastic should be evaluated, and a supplemental diet of organic feed and plastic needs to be considered.

Here, we propose that species such as cockroaches could feed on and/or degrade plastic and could offer the solution to meet UN sustainable development goals to eliminate plastic pollution. The investigation could start using grocery plastic bags and/or microplastics such as low‐density polyethylene which is already known to be reduced in size by gut bacteria from other insects (Lwanga et al., 2018). This will address plastic pollution which remains a huge concern worldwide as the contamination of aquatic habitats poses a challenge to ecosystems (Trotter et al., 2019). Recent reports estimate that at least 79 thousand tonnes of ocean plastic are afloat inside an area of 1.6 million km^2^ inside the ‘Great Pacific Garbage Patch’, which is much greater than previously reported, highlighting the need to identify novel methods to recycle or eradicate plastic pollution (Lebreton et al., 2018). Moreover, synthetic plastic is either non‐biodegradable or degrades extremely slowly, for example, polythene requires approximately 1000 years to degrade (Lebreton et al., 2018; Sangale et al., 2019). Plastic waste has also been highlighted by the 17 sustainable goals adopted by the United Nations (UN SDGs). Several goals are relevant to reducing and/or eliminating plastic pollution, such as Goal 11 (sustainable cities and communities), Goal 12 (responsible consumption and production), Goal 14 (life below water) and Goal 15 (life on land) (https://sdgs.un.org/goals). At present, various strategies to dispose of plastic waste are in place including landfill sites, incineration, recycling, production of fuel and degradation or biodegradation (Sangale et al., 2019). However, these strategies can be expensive, and require infrastructure; and may not be possible for developing countries to put them into place easily and effectively. Here, we propose a cheap and effective bio‐resource, which does not require investment and can be set up relatively easily, even in the developing countries at the community level in rural areas.

Notably, *Homo sapiens* are just one of the species amongst millions of others, and in comparison, are a relatively new addition to the planet. On the other hand, species such as cockroaches have shown the ability to adapt, evolve and survive successfully over millions of years, and thus we ought to learn from these species. Even with exposure to stressful surroundings and hazardous materials, these species thrive under conditions that are considered harmful to *H. sapiens*. Here, we propose to utilise these hardy insects to confront the menace of plastic pollution and study the feeding ability of cockroaches on various types of plastics. This hypothesis‐determined research inquiry is relevant and interesting as it is imperative to utilise pests such as cockroaches, one of the hardiest insects, for a positive task in eliminating or reducing plastic waste. The use of insects is a novel concept in biomedical investigation, but it offers outstanding opportunities to utilise what is one of the most hated insects on the planet for a good cause, which is to manage a growing amount of plastic waste worldwide. For example, setting up plastic recycling plants involves land costs, building costs, equipment (machinery) costs, human resources, managing costs, running costs such as electricity, and other utilities associated with the collection of plastic. Depending on the volume and type of the project and the number of employees, the approximate costs associated with setting up a recycling plant can vary, often running into millions of dollars (Jamasb & Nepal, 2010). In the absence of any incentives, this is neither feasible nor profitable for the majority of developing countries. For example, many areas in developing countries lack infrastructure or suffer from routine and prolonged power cuts making such initiatives impractical. For such communities, there is a need to develop cost‐effective, and small‐scale options with minimum requirements that can be implemented at the regional and household level. The proposed use of cockroaches to eliminate plastic is a promising avenue that ought to be explored as an alternative to existing technologies. For example, composting food waste at home is a natural way to decompose food waste and other organic materials and is relatively easy to set up. A similar system that could house cockroaches in a hatch or cage could be proposed at the household level and would be a valuable addition to communities in developed countries as well, where the collection of plastic involves local and federal legislation, is costly, and/or lacks incentive. One or two cockroaches (based on our preliminary observations in the laboratory) could be utilised per gram of plastic waste, and cages are relatively easy to set up, which should be sealed to ensure limited/negligible effects on the surrounding ecosystem, with access to water for cockroaches' nutritional requirements, and allow for light penetration, as cockroach circadian rhythms follow a 12‐h light and dark pattern (Ali et al., 2017; Weihmann et al., 2015). Other household waste items comprising cellulosic material (such as food packaging and as well as other types of food waste) may also be added, as these are well‐recognised to be consumed by cockroaches (Ali et al., 2017). Nonetheless, the system will need to be evaluated carefully, in various scenarios, to ensure safety and prevent escape of cockroaches before being utilised, given their potential role in the transmission of pathogens (Nasirian, 2019). A factor to consider will be the number of cockroaches needed per tonne of plastic, which may require 1–2 million cockroaches. Although, it is anticipated that the increase in cockroach number may result in increased elimination of plastic waste, their numbers can be regulated due to their cannibalistic tendency in a particular space (i.e., cage) to manage food and population size, however it needs to be evaluated further. Furthermore, it would be interesting to undertake future studies on investigating the role of the gut microbiota of the cockroach, their contribution to the elimination of plastics and the underlying molecular mechanisms, as well as to evaluate how plastic pollutants are managed by these resilient species, alongside the development of a prototype for a household system. The use of cockroaches has been suggested to comprehend the toxicological mechanisms or detection of environmental contamination to provide solutions for both humans and animals (Adedara et al., 2022). However, possible negative effects on the cockroach will need to be determined; as previous studies indicate that when earthworms digested microplastics, changes in the composition of their gut microbiome were seen as well as in springtails (Rodriguez‐Seijo et al., 2017; Zhu et al., 2018). This includes the notion that cockroaches may be responsible for the transmission of bacteria such as *Salmonella* and other bacterial contaminants, or may cause allergies in susceptible people, with low levels of tolerance against these insects (Patel & Meher, 2016; Turner et al., 2022; Wang et al., 2019). Nonetheless, the robust immune system and the gut microbiome of cockroaches may offer tremendous opportunities to carry out future studies to identify novel molecules to solve age‐old problems of countering contaminants, such as the elimination of plastic. This is in addition to other solutions to produce bio‐based plastic‐like sustainable and biodegradable polymers that have the potential to replace plastics and has potential to contribute to the UN SDGs (Mukherjee & i Koller, 2022). All in all, cockroaches can endure high radiation, flourish in unsanitary circumstances, thrive on germ‐infested feed, and can even digest organic polymer cellulose, hence they offer potential resources to eliminate plastic pollution. Although the problem of plastic pollution is immense and we do not suggest that this problem can be solved by a single type of solution, this is a timely and topical area of research to use cockroaches as a model organism in biomedical investigation to support UN sustainable development goals. Currently, many insectaries in universities house a variety of insects such as mosquitos, locusts, and cockroaches, and the design of these may be incorporated and adapted for use in plastic pollution elimination, as well as to prevent any potential escape of cockroaches (Koehler et al., 1994). Alternative insect models such as the black soldier fly could also be considered (Cho et al., 2020). Intensive future research is needed to realise these expectations.

## AUTHOR CONTRIBUTIONS

**Ruqaiyyah Siddiqui:** Conceptualization (equal); formal analysis (equal); resources (equal); writing – original draft (equal). **Naveed Ahmed Khan:** Conceptualization (equal); formal analysis (equal); project administration (equal); resources (equal); writing – original draft (equal).

## CONFLICT OF INTEREST STATEMENT

The authors declare no conflicts of interest.

## Data Availability

No new data were generated or analysed in support of this research.
